# Posttraumatic Growth Among Siblings Bereaved by a Drug-Related Death: A Mixed-Method Study

**DOI:** 10.3390/bs16040549

**Published:** 2026-04-07

**Authors:** Monika Alvestad Reime, Liv Marit Kleppe, Nina Bringedal, Kristine Berg Titlestad

**Affiliations:** 1 HVL Business School, Faculty of Technology, Environmental and Social Sciences, Western Norway University of Applied Sciences, 6805 Sogndal, Norway; 2 Department of Welfare and Participation, Faculty of Health and Social Sciences, Western Norway University of Applied Sciences, 6805 Sogndal, Norway; liv.marit.kleppe@hvl.no (L.M.K.); nina.bringedal@hvl.no (N.B.); kbti@hvl.no (K.B.T.)

**Keywords:** siblings, post-traumatic growth (PTG), drug-related death, bereavement, self-efficacy, social support, mixed-methods

## Abstract

Losing a sibling to a drug-related death can lead not only to profound grief but also to unexpected psychological growth. This mixed-method study examined such growth among siblings bereaved by a drug-related death in Norway, combining survey data from 78 participants with interviews from ten siblings. Quantitative findings showed that appreciation of life and personal strengths were the most prominent domains of growth. Regression analysis indicated that self-efficacy explained most of the variance in growth when controlling for time since death, whereas social support did not make a unique contribution. Qualitative findings added depth by revealing how growth was experienced through closer family relationships and a heightened sense of empathy toward people in vulnerable situations. These accounts suggest that growth may involve a reorientation of values and deeper relational ties, aspects that standardized measures may not fully capture. Although based on a small and relatively homogeneous sample, the integrated results point to the importance of internal coping resources and family connectedness in fostering growth after a stigmatized loss. Further research should explore these mechanisms in more diverse populations and examine how they evolve over time.

## 1. Introduction

The traumatic loss of a sibling to a drug-related death is a painful life event, undoubtedly negatively affecting the lives of the bereaved persons left behind (Stroebe et al., 2024). While traumatic bereavement following sudden and unnatural deaths is widely associated with severe distress (see, e.g., Buur et al., 2024; Djelantik et al., 2020), research increasingly highlights that positive psychological changes can also emerge in the aftermath of a traumatic event (Calhoun et al., 2010; Henson et al., 2021; Hurst & Kannangara, 2024; Levi-Belz et al., 2021; Michael & Cooper, 2013). Posttraumatic growth (PTG) refers to these positive changes that occur as individuals struggle with stressful and traumatic circumstances, such as the loss of a close person (Calhoun et al., 2010; Tedeschi & Calhoun, 2004). Importantly, PTG results from the struggle with the trauma, not from the event itself (Henson et al., 2021). PTG has been conceptualized both as a parallel process and as a counterpart to post-traumatic stress disorder, which involves severe symptoms such as intrusive flashbacks (Hurst & Kannangara, 2024). The coexistence of personal distress and growth underscores the complexity of PTG and provides a critical context for exploring this phenomenon (Calhoun et al., 2010; Tedeschi & Calhoun, 2004).

Tedeschi and Calhoun’s (2004) model of PTG outlines five domains of positive change that may occur as individuals struggle with traumas: improved relationships; new possibilities in life; greater appreciation of life; enhanced personal strength; and spiritual development. However, PTG is not experienced by everyone. Prior evidence syntheses have highlighted key factors associated with PTG, including coping strategies, social support, cognitive processing and rumination, religiosity/spirituality, and personality traits (Henson et al., 2021; Michael & Cooper, 2013). Opportunities for PTG have also been linked to the cause and context of death, as well as the relationship to the deceased (see, e.g., Johnsen & Afgun, 2021). This article analyzes and integrates quantitative and qualitative evidence to understand pathways to PTG among siblings bereaved by a drug-related death.

### 1.1. Drug-Related Deaths

Drug-related deaths are a major global health concern. In the United States (US), the increasing rates of drug-related deaths in recent decades are described as an epidemic that severely influences thousands of bereaved family members and friends left behind (U.S. Centers for Disease Control and Prevention [CDC], 2025). For every deceased person, it can be estimated that there will be around ten to 15 persons bereaved (Dyregrov et al., 2020). In 2023, 105,007 drug overdose deaths were registered in the US, with an age-adjusted rate of 31.3 deaths per 100,000 standard population (U.S. Centers for Disease Control and Prevention [CDC], 2025). Other deaths related to the use of illegal substances come on top of this, such as deaths by illness, accidents, suicide, and violence. In Europe, the rates for drug-related deaths are also alarming, with a minimum estimate of 7500 drug-induced deaths in 2023, representing 24.7 deaths per million population aged 15 to 64 (European Union Drugs Agency [EUDA], 2025).

Research from the US and Norway indicates that people bereaved by a drug-related death are at a particular high risk for developing severe bereavement complications, elevated levels of prolonged grief (PGD) symptoms (Bottomley et al., 2022; Titlestad & Dyregrov, 2024), post-traumatic stress disorder (see American Psychiatric Association, 2013; Bottomley et al., 2022), impaired social health (Kalsås et al., 2023), severe depressions (Bottomley et al., 2022), and even increased mortality (Christiansen et al., 2020). These vulnerabilities have been linked to the sudden and unnatural nature of the death, the circumstances surrounding the death, and the period preceding the death (Kalsås et al., 2023; Titlestad et al., 2021a, 2021b), and the stigma and disenfranchisement that often follows deaths related to the use of illegal substances (Bottomley et al., 2025; Reime et al., 2026; Titlestad et al., 2021b; Valentine et al., 2016). This stigma is frequently linked to the perception that such deaths may be self-inflicted (Doka, 1999) and to the illegal nature of drug use, which is often viewed as a criminal activity (Kheibari et al., 2022; Valentine et al., 2016). The concept of associated stigma is pertinent here, as it describes the situation in which bereaved individuals identify with or are associated with the stigma attached to the deceased person (Sheehan & Corrigan, 2020).

### 1.2. Stigmatized Bereavement and PTG

Research has come to inconclusive findings regarding the relationship between stigmatized deaths and PTG. For example, a meta-analysis of research on suicide bereaved individuals showed no significant difference in the prevalence of PTG between suicide bereaved individuals and other groups of bereaved individuals (sudden and expected deaths) (Levi-Belz et al., 2021). Furthermore, the meta-analysis showed that the bereaved individuals most frequently reported PTG in the domains of “Relating to others”, “Spiritual change”, and “Appreciation of life”. Among variables identified as significantly associated with PTG were time since death, religious and spiritual involvement, positive and adaptive coping styles, and self-disclosure and social support (Levi-Belz et al., 2021).

A few studies have explored PTG in individuals bereaved by a drug-related death. From the same project as the present article, Titlestad et al. (2022) examined paths to PTG in parents bereaved by a drug-related death (*n* = 89). While the quantitative findings showed the highest score on the domain “personal strengths” and the lowest score on “new possibilities”, the qualitative data showed recognition of new paths in life to be important for many of the interviewees. High levels of self-efficacy and social support were positively associated with PTG (Titlestad et al., 2022). Self-efficacy can be understood as a personality resource influencing how people evaluate their capabilities, and consequently their choices of actions and environments (Bandura, 1982). Individuals who hold strong beliefs in their capacity to effect change tend to be more proactive when confronting challenging life circumstances. In bereavement theory, meaning-making has been identified as a central process for healthy adaptation to loss (Gillies & Neimeyer, 2006; Neimeyer, 2019). For example, in the Dual Process Model of bereavement, the search for meaning serves as a key motivation driving adaptive oscillation between loss- and restoration-oriented (Stroebe & Schut, 2001). Meaning-making denotes the active process by which a bereaved person seeks to make sense of the loss, encompassing activities of meaning reconstruction such as renegotiating the relationship with the deceased, searching for compensatory benefits, and revising one’s identity (Gillies & Neimeyer, 2006).

The importance of social support and open dialogue about the loss is also supported in qualitative studies (Feigelman et al., 2020; O’Callaghan et al., 2023). For example, O’Callaghan et al. (2023) find peer support groups to be perceived as particularly helpful among family members bereaved by a drug-related death. However, not all interviewees found support groups helpful, but rather perceived grief as something to be dealt with internally within the family (O’Callaghan et al., 2023). Social support has been defined as “verbal and non-verbal communication between recipients and providers that reduces uncertainty about the situation, the self, others, or the relationship, and functions to enhance the perception of personal control over one’s life experiences” (Albrecht & Adelman, 1987, p. 19). Other definitions emphasize the role of social support in shaping an individual’s self-perception, highlighting experiences of being cared for, loved, esteemed, and valued (Cobb, 1976).

Quantitative studies have identified several factors positively associated with PTG in the context of drug-related bereavement, including self-compassion (Sperandio et al., 2022), self-forgiveness (Sperandio et al., 2023), as well as active emotional coping and problem-focused coping strategies (Hill & O’Brien, 2024). In a recently published review on studies on PTG in the case of a drug-related death, Jean-Berluche (2024) sums up three main mechanisms that have shown to facilitate PTG: engaging in open dialogue with supportive communication, fostering self-compassion and hope, and reframing loss (meaning-making).

While only a few studies in recent years have examined PTG among individuals bereaved by drug-related deaths, there remains a need for research that explores the grief-related consequences for the diverse individuals who experience such traumatic bereavement each year (Hill & O’Brien, 2024). Research on PTG has concentrated either on examining PTG among parents (Feigelman et al., 2020; O’Callaghan et al., 2023; Titlestad et al., 2022), or they have addressed PTG in bereaved individuals generally without focusing on the relation to the deceased (Hill & O’Brien, 2024; Sperandio et al., 2022, 2023).

### 1.3. Siblings and PTG

The sibling relation is one of the longest-lasting and closest of familial relationships, but has gained surprisingly little attention in bereavement research (Davidson, 2018; Rostila et al., 2019, 2012). The loss of a sibling in childhood has been associated with risk factors such as increased mortality and mental health problems (Bolton et al., 2016; Rostila et al., 2019; Yu et al., 2017). Also, research has shown a tendency for siblings to protect their parents and thereby prioritize their parents’ grief before their own (Adams et al., 2019; Funk et al., 2018). In a culturally constructed hierarchy of grief, siblings tend to view themselves as less legitimate grievers (Davidson, 2018; Meen et al., 2025; Robson & Walter, 2013). When death is stigmatized, the risk for bereavement complications for the bereaved sibling can be increased. This has, among other things, been related to reduced access to social support following the taboos that surround stigmatized deaths, leading to self-isolation and others’ withdrawal (Dyregrov et al., 2025; Meen et al., 2024; Perrin, 2022).

Despite the distress and pain following from the loss of a sibling, some researchers have also explored the personal growth that can follow from the loss of a brother or sister (Adams et al., 2019; Johnsen & Afgun, 2021). For example, Adams et al. (2019) found from a qualitative study of seven siblings bereaved by suicide that the siblings experienced strengthening their coping resources, and recognized that they could use their own experience to help others. Johnsen and Afgun (2021) examined PTG and its relation to some associated variables (PGD, gender of the bereaved, and relationship to the deceased) among bereaved siblings (*n* = 43) and close friends (*n* = 86), following the 2011 Utøya mass shooting in Norway. The results showed no association between PTG (measured by the “Post-traumatic Growth Inventory-Short Form, PTGI-SF”) and symptoms of PGD, but females reported higher levels of both PTG and symptoms of PGD compared to males (Johnsen & Afgun, 2021). To date, we have not identified studies that examine PTG among siblings bereaved by a drug-related death. This article aims to address this gap in the literature.

### 1.4. Research Question and Hypothesis

The use of mixed methods can provide nuanced insights into the complexity of PTG among siblings bereaved by a drug-related death and deepen understanding of the mechanisms associated with PTG and its individual impact. This study explores pathways to PTG in this population, combining data from a quantitative survey and qualitative interviews. Quantitative findings will be elaborated upon using qualitative data to enrich interpretation.

The following research question guides the quantitative analysis: To what extent do siblings bereaved by a drug-related death experience positive changes as measured by the PTGI-SF scale, and what is the association between time since death, social support, and perceived self-efficacy with levels of PTG? Based on prior literature illustrated in the introduction section, three hypotheses are formulated:

1. Time since the sibling’s drug-related death will be positively associated with overall PTG;
2. Higher levels of perceived social support will be positively associated with overall PTG;
3. Higher levels of perceived general self-efficacy will be positively associated with PTG.

The qualitative analysis addresses the question: How do siblings bereaved by a drug-related death experience positive changes following the loss? Finally, the mixed-methods question is: How can pathways to PTG among siblings bereaved by a drug-related death be understood through the integration of quantitative and qualitative data?

## 2. Materials and Methods

This mixed-method study is part of a larger mixed-method project on drug-death bereavement, the Drug-related bereavement and recovery project (the END project). The END project aimed to explore the consequences of losing a close one to a drug-related death, and experiences with support, both from the bereaved persons’ perspectives and helpers’ perspectives. In total, 255 bereaved persons (i.e., parents, children, siblings, other relatives, and friends/partners) and 105 helpers participated in the project.

### 2.1. Recruitment and Sample

This article draws on data from the sibling subsample of the END project, and includes survey responses from siblings who completed items about PTG (*n* = 78) and in-depth interviews with ten bereaved siblings from the survey sample. The survey was conducted in 2018 using a convenience sampling strategy. Recruitment was carried out through multiple channels, including flyers emailed to all Norwegian municipalities, personal contact with key personnel in municipal services, health and welfare services, drug treatment services, the Norwegian Labor and Welfare Administration, and civil society organizations. Additional recruitment strategies included social media advertising and snowball sampling. No upper limit was set for time since death, but persons bereaved within the last three months were excluded. Interview participants were selected from the survey sample using a purposive sampling strategy to ensure variation in background characteristics, including time since death, age, gender, and place of residence.

### 2.2. Sample Characteristics

The survey sample (Table 1) consisted primarily of sisters (*n* = 69; 88.5%). The average age of participants at the time of the survey was M = 39 years. The mean time since death was M = 112 months (9.3 years), with a range of 3–360. The deceased siblings had an average age of M = 31 years at the time of death. Participants reported having a relatively close relationship with their sibling, with a mean score of M = 1.55 on a Likert scale ranging from 1 (very close) to 5 (not close). Educational attainment was high: 49 participants (62.8%) had completed higher education (college/university), compared to 37.9% in the general Norwegian population (Statistics Norway [SSB], 2026b). More than half of the siblings were employed full-time (*n* = 48; 61.5%), and most reported having some form of employment (full-time, part-time, or sick leave) (*n* = 62; 79.4%). Regarding annual household income, 25 respondents (32.1%) reported earning between 750,000–999,999 NOK, which is above the median household income in Norwegian (Statistics Norway [SSB], 2026a).

The interview sample (Table 1) consisted of ten siblings, seven of whom were women. The average time since death was M = 114 months (approximately 9.5 years), with a range of 9–216. The average age of the bereaved sibling at the time of the interview was M = 43 years, while the deceased siblings had an average age of M = 34 years at the time of death. Participants reported being close to their sibling at the time of death, with a mean score of M = 1.7 on a Likert scale ranging from 1 (very close) to 5 (not close). Educational attainment was high, with eight participants having completed higher education and two having completed senior high school. At the time of the interview, eight siblings were employed full-time, one was studying, and one reported “other”. Annual household income ranged from 250,000 NOK to 1,250,000 NOK.

### 2.3. Mixed-Method Design

When considering the combination of qualitative and quantitative data, various strategies for mixed-method designs can be employed (Johnson & Christensen, 2024). This study is based on a concurrent design with a larger quantitative component supplemented with interview data.

### 2.4. Measurements

The survey consisted of 22 background variables and 87 items from validated measurements. For this article, we have utilized different background variables, the Post-traumatic Growth Inventory-SF (PTGI-SF) instrument, the Crisis Support Scale (CSS), and the General Self-Efficacy Scale-SF (GSF-SF).

#### 2.4.1. Post-Traumatic Growth Inventory-SF (PTGI-SF)

The PTGI-SF scale is a 10-item questionnaire originally developed in the US for American samples. It comprises five subscales, each rated on a six-point Likert scale reflecting the degree of personal change experienced as a result of the death, with higher scores indicating a higher level of perceived change (Cann et al., 2010; Tedeschi & Calhoun, 2004). In the present study, four subscales were assessed: “Relation to others”; “New possibilities”; “Personal strengths”; and “Appreciation of life”. The fifth subscale, “Spiritual change”, was excluded based on cultural validity considerations (see Blix et al., 2015). The Cronbach alpha (α) for the eight-item scale used in this study was 0.888, indicating good internal consistency. Subscale reliability was acceptable: Relations to Others (α = 0.738), New Possibilities (α = 0.784), Personal Strengths (α = 0.788), and Appreciation of Life (α = 0.799).

#### 2.4.2. The Crisis Support Scale (CSS)

The CSS is a seven-item measure of perceived social support rated on a seven-point Likert scale (Elklit et al., 2001; Joseph et al., 1992). The five first items measure perceived positive support, with higher scores indicating higher levels of support. Internal consistency for the five-item version used in this study was acceptable (α = 0.758).

#### 2.4.3. The General Self-Efficacy Scale-SF (GSE-SF)

The GSE-SF consists of five items assessing optimistic self-belief regarding successful coping with a variety of adverse life situations (Schwarzer & Jerusalem, 1995). The five items are rated on a four-point Likert scale, from 1 (“Not at all true”) to 4 (“Exactly true”). The Norwegian version of the GSE-SF has been validated by Tambs and Røysamb (2014). Internal consistency for the five-item scale was good (α = 0.861).

### 2.5. Qualitative Interviews

In-depth interviews with siblings were conducted between 2018 and 2019 by three researchers from the END project. Interviews took place either in the participants’ homes or another preferred location and lasted between one and a half and three hours. A thematic interview guide was developed, covering five main topics: (1) time before death; (2) time after death; (3) stigma from surroundings and self-stigma; (4) formal and informal help and support; and (5) self-coping mechanisms and posttraumatic growth, including the question: “Have you experienced any changes that brought something new or contributed to personal growth after the death?”. All interviews were audio-recorded and fully transcribed. As the interviews were conducted in the participants’ native language, quotations presented here were translated by the first author for this article. In total, the transcripts consisted of 233 single-spaced pages.

### 2.6. Ethics

The END project was approved in February 2018 by the Norwegian Regional Ethical Committees for Medical and Health Research Ethics (2017/2486/REK vest). The project adheres to the ethical principles outlined in the Declaration of Helsinki and complies with national and international regulations regarding participant privacy (e.g., the Norwegian General Data Protection Regulation and guidelines from the Regional Ethical Committee). Data are securely stored on the university’s research server.

All participants received written information about their rights, study content, and procedures, and provided informed consent. They were assured full anonymity and informed of their rights to withdraw at any time. Interviewing participants about bereavement requires sensitivity and careful attention to ethical considerations. Participation may pose a risk of distress, as interview topics can reactivate painful memories or rumination about the death. However, research participation under protective circumstances may also offer benefits, such as providing an opportunity to express emotions and share experiences, which can be perceived as meaningful and helpful to others (Beck & Konnert, 2007). In this study, the research team took steps to ensure a safe and supportive interview environment. Participants chose the interview location and could request breaks at any time. After the interview, they were informed about the option to contact the project manager if they needed further support, but no such contact was made.

### 2.7. Analysis

#### 2.7.1. Quantitative Analysis

Mean scores were calculated for the overall PTGI-SF scale, four subscales, and the eight individual items. Mean scores were also computed for the overall CSS positive support scale (five-items) and for the GSE-SF scale. Bivariate correlations using Pearson’s product-moment coefficient (*r*) assessed associations between time since death and PTG, social support (total score) and PTG, and self-efficacy and PTG. Potential predictors of PTG were examined in a hierarchical multiple regression analysis. Time since death was entered as a control variable in the first step, while the social support and self-efficacy were entered in the second step.

Effect sizes were evaluated using the coefficient of determination (*R*^2^), and squared semi-partial correlations (*sr*^2^) were used as effect size measures for the linear regression. *R*^2^ indicates the proportion of variance in PTG explained by the combined predictors, whereas *sr*^2^ reflects the unique contribution of each predictor. The quantitative analysis was conducted by the first author (MAR) and verified by the last author (KBT) to ensure methodological rigor.

#### 2.7.2. Qualitative Analysis

Qualitative data were analyzed using reflexive thematic analyzes, which provides a systematic yet flexible approach to coding and theme development (Braun & Clarke, 2022). In the first phase, three authors (LMK, NB, and MAR) read and re-read all ten interview transcripts to become familiar with the data. Each author independently took notes and identified quotes relevant to the research question that could illustrate patterns of meaning (see Braun & Clarke, 2022). Based on these notes, initial codes were developed separately by each author. During this process, the authors noted that descriptions of experiences of growth were limited. However, the depth of the interviews allowed us to identify tendencies in how participants experienced growth.

Next, codes were reviewed and grouped into potential themes. Two authors (LMK and MAR) compared notes and collaboratively developed two preliminary themes with subordinate codes, which were then compared with notes from the third author (NB). The empirical data were re-read, and themes were refined through discussion, guided by the PTGI-SF dimensions used in the survey.

#### 2.7.3. Mixed Analysis Matrix

The two datasets were first analyzed separately, and the results were visualized using a data display strategy (Johnson & Christensen, 2024). In the next step, qualitative and quantitative descriptive data were integrated, and presented in a joint data display (Johnson & Christensen, 2024). 

## 3. Results

### 3.1. Quantitative Results

**Figure 1:**
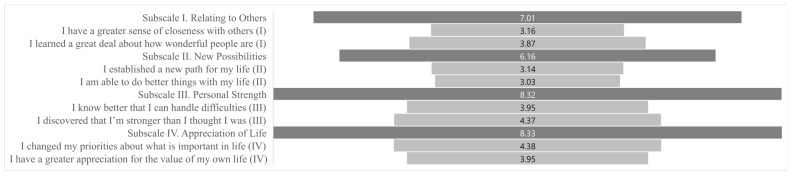
Mean scores for the four PTGI-SF subscales and their corresponding items.

The siblings’ average PTGI-SF sum score was M = 29.77 (SD 9.65), with sisters scoring M = 29.8 and brothers M = 29.56. Scores ranged from 11 to 48. The highest item scores were for “I changed my priorities about what is important in life” (Appreciation of Life; M = 4.38) and “I discovered that I’m stronger than I thought I was” (Personal Strengths; M = 4.37). The lowest item score was for “I am able to do better things with my life” (New Possibilities; M = 3.03). At the subscale level, the highest mean scores were for “Appreciation of life” (M = 8.33) and “Personal strengths” (M = 8.32), while the lowest was for “New possibilities” (M = 6.16) (see Figure 1).

Pearson’s product-moment correlations (*r*) were calculated to assess relationships between all study variables and PTG (see Table 2).

Hierarchical multiple regression analysis showed that time since death explained 5.7% of the variance in PTG when entered in the first step [*R*^2^ = 0.057, F(1, 75) = 4.713, *p* < 0.05]. In the second step, social support and self-efficacy explained an additional 15.8% of the variance in PTG [Δ*R*^2^ = 0.158, F(2, 73) = 7.390, *p* < 0.01]. Social support did not contribute significantly, whereas general self-efficacy accounted for most of the unique variance (*sr*^2^ = 0.348, 34.8%, *p* < 0.01), followed by time since death (*sr*^2^ = 0.224, 22.4%, *p* < 0.05) (see Table 3). Overall, the model explained for 21.8% of the variations in PTG.

### 3.2. Qualitative Results

Growth in relation to others was constructed as the main theme. This theme was supported by two codes (Table 4), which are presented in this section together with illustrative interview excerpts.

#### 3.2.1. Greater Sense of Closeness with Family Members

Some of the siblings described how the death strengthened family bonds and increased their sense of closeness with family members. The loss appears to have altered family relations and dynamics, as illustrated by this brother:

I remember we used to have a lot of deep conversations like that, which I don’t think we’ve had much of in our family (before the death), talking about what really matters and what we do now. How do we relate to this and so on? It has affected the dynamics in our family. After he passed away, there have been some concrete changes, like now we have started hugging each other every time we meet; we rarely did that before (…) that it’s our deceased brother’s merit that we have become much closer and that we are more physical with hugs and such.

(ID 54)

This account illustrates both emotional and physical changes in family interactions. Conversations are perceived as more honest and profound, signaling increased closeness. The introduction of hugging reflects a shift toward greater comfort with intimacy. Other examples in the data also indicate changes in interactional patterns, such as this sister describing how the frequency of contact between family members has increased following her brother’s death:

In relation to family, we were very much together for a long time after the death, and I still think we spend a lot of time together compared to other families (…) we meet very often at my parents’ place and that has been very important. You don’t necessarily have to talk about the death itself, but just being together and talking about him sometimes, in a way he’s still with us, what would he be doing now? Or what would he say now, for example? Yes. That’s important, and it’s still important.

(ID 24)

In this instance, the family appears to create an emotional space where memories can be shared and disclosed, which can contribute to increased connectedness and enhance the sense of closeness among family members, allowing for mutual support and recognition.

#### 3.2.2. Increased Compassion Towards Others in Vulnerable Life Situations

Across interviews, participants emphasized a growing sense of compassion towards individuals in vulnerable circumstances, including people with substance use problems, and others facing significant life challenges. They described developing greater respect for diverse life trajectories and a deeper understanding of both the resources and difficulties these individuals encounter. This shift is illustrated by this sister:

Something I’ve seen more and more as I get older, is that I have become much more empathetic. Not that I was very unempathetic before, but that people are so different, and we need to accept that people, the things they do, because no one is just one thing or another. For example, a deeply depressed person has many positive sides and doesn’t just walk around being gloomy. A substance abuser has many good qualities and a positive history; the addiction is just one aspect of them. So I hope and believe that I have become a bit more tolerant of the fact that life can take some turns that may cause someone not to behave well all the time, but that doesn’t define the whole person.

(ID 31)

This reflection highlights increased empathy, patience, and tolerance, though the participant acknowledges that aging may also play a role. Others emphasized recognizing the deceased sibling’s significance in others’ lives, as illustrated by another sister:

And then suddenly you saw that, well, drug addicts have a network, (mhm) he was much more than just a substance abuser; he had many friends, there were children of friends who gave speeches at his funeral. I have never been to a funeral with so many people, and it was a new experience. It made me feel, I felt a bit of joy almost that he had lived a life (…) he wasn’t just a burden to everyone, he wasn’t just a source of sorrow. He had a life; he meant something to many people, eh, yes. There was a kind of goodness in that. So I think that’s maybe what I have brought with me moving forward, that (…) none of us live in vain. That is, he actually had a life, and that was something.

(ID 86)

This participant’s account suggests that recognizing the value of her deceased sibling’s life fostered greater compassion toward others in similar circumstances. Her reflection conveys a sense of humility about human diversity and the different paths people take, underscoring the importance of acknowledging and respecting these differences.

### 3.3. Mixed-Methods Results

Table 5 presents the joint display used to integrate quantitative and qualitative findings, by systematically aligning quantitative results with qualitative themes and illustrative quotes. This clarifies how the two strands interface and where qualitative insights elaborate on the quantitative patterns. The quantitative strand indicated that siblings experienced PTG across multiple domains, with the most pronounced changes related to appreciation of life and personal strength, followed by relational growth and new possibilities. The qualitative strand complements these findings by illustrating how growth was expressed in everyday life. Participants described strengthened family bonds, more open conversations, and increased physical closeness. They also reflected on becoming more compassionate toward individuals in vulnerable circumstances and developing a deeper respect for diverse life paths. These accounts mirror the most prominent domains in the quantitative data and highlight a reorientation of values, captured in the notion of changed priorities. Patterns suggest that internal resources such as self-efficacy and the passage of time may be associated with these changes, aligning with qualitative accounts of openness and intimacy. Together, these findings indicate that enduring growth is shaped by meaning-making and internal coping resources rather than acute crisis support, manifesting as relational intimacy, broader value-based perspectives, and changed life priorities.

## 4. Discussion

This study was set up to deepen the understanding of pathways to PTG among siblings bereaved by a drug-related death through the integration of qualitative and quantitative data. The integration indicates a coherent picture in which the most pronounced quantitative changes are Appreciation of life and Personal strength, while the qualitative strand illuminates how these changes are enacted in everyday life through strengthened family bonds and increased compassion toward others. Taken together, the pattern suggests that PTG in this context is both individual and relational, reflecting a value-based reorientation and identity reconstruction in the wake of stigmatized loss. The regression findings lend tentative support to this interpretation: self-efficacy, an internal coping resource (Bandura, 1982), explained most of the variance in PTG (controlling for time since death), whereas crisis support did not contribute uniquely despite its correlation with PTG. This divergence underscores that enduring growth may depend more on internal resources and meaning-making processes than on acute support, while qualitative accounts nevertheless show that relational closeness within families remains central to how growth is lived and sustained. Hence, we discuss the unique position of siblings bereaved by a drug-related death within this bereavement context and consider how growth, particularly positive changes in family relationships, can be understood against the backdrop of stigmatized loss. Furthermore, we explore the role of self-efficacy, which emerged as a key factor significantly associated with the PTG experiences in the sibling population.

### 4.1. Positive Changes in Family Relations

Consistent with previous research on traumatic bereavement, our findings highlight improved relations as an important aspect of PTG, aligning with results in a systematic review of suicide bereavement (Levi-Belz et al., 2021), and a study of siblings and close friends after the mass attack at Utøya (Johnsen & Afgun, 2021). These parallels suggest that sibling bereavement shares common features with other traumatic losses in fostering positive psychological change.

Two relational improvements were evident in the qualitative analysis: siblings described becoming closer to other family members (both parents and other siblings), and developing a greater understanding of people in vulnerable life situations. These changes reflects the theory of PTG which emphasizes that growth in relationships can involve deeper, more meaningful connections and heightened compassion (Tedeschi & Calhoun, 2004). As Calhoun et al. (2010) note, an increased sense of connectedness with others can extend to empathy for others, an observation echoed in our data. For example, Calhoun et al. (2010, p. 128) refer to one bereaved person saying: “I’ve become very empathetic towards anybody in pain and in any kind of grief”. The reference to “anyone” is also supported by results from our study. Unlike parents interviewed in the same project as the present (Titlestad et al., 2022), who described compassion primarily toward individuals using drugs, siblings described a broader sense of empathy.

Notably, all qualitative examples of relational growth in relation to others found in this study suggest that grief was largely contained within the family system. For some, this shared struggle fostered closeness, a pattern also observed among bereaved parents in the same project (Titlestad et al., 2022). Additionally, the study by O’Callaghan et al. (2023) found that some participants preferred to keep their grief within the family. PTG theory similarly posits that increased closeness typically occurs with significant others such as family or close friends (Calhoun et al., 2010). Findings suggesting that grief was kept within the family, can indicate a lack of other supportive relations. The stigma associated with illegal drug use (see, e.g., Bottomley et al., 2025; Jean-Berluche, 2024; Reime et al., 2026; Titlestad et al., 2021b) is relevant to the present discussion. Bereaved siblings may be socially discredited or excluded through association with a deceased sibling’s lifestyle, reflecting societal stigma (Sheehan & Corrigan, 2020). Alternatively, siblings may internalize this societal stigma, which can negatively affect their self-perception and social functioning. This can result in both self-isolation and withdrawal from others, diminishing the opportunity to maintain meaningful relationships following the death (Doka, 1999; Dyregrov & Selseng, 2022; Reime et al., 2026; Valentine et al., 2016).

While keeping traumatic deaths and grief within the family can promote psychological growth for some siblings if the family is coping with the loss in adaptive ways, it may also leave siblings vulnerable. Hierarchies of grief often prioritize parents’ loss, positioning siblings as less legitimate mourners (Davidson, 2018; Gilbert, 1996; Meen et al., 2024, 2025; Peskin, 2019; Robson & Walter, 2013). Additional barriers include difficult family relationships, harmful upbringing conditions, and exhaustion linked to pre-loss circumstances (Dyregrov et al., 2025). Our findings highlight the need for a nuanced approach to supporting positive psychological changes in relationships, one that strengthens family dynamics and communication while also fostering connections beyond the immediate family network.

### 4.2. Self-Efficacy—A Path to PTG Among Siblings?

Our findings indicate that self-efficacy is the variable most strongly associated with variations in PTG, a result consistent with the study of bereaved parents from the same project (Titlestad et al., 2022). These results align with the results from Sperandio et al. (2022), who reported that an emotionally positive self-attitude predicted significant PTG among individuals bereaved by a drug-related death. A positive self-view or confidence in one’s own resources can be viewed as a way of making meaning of one’s situation and resources in light of the loss (Gillies & Neimeyer, 2006; Neimeyer, 2019). Within the PTG model, challenges to peoples’ assumptive world-views and threats to ones’ self are perceived as a starting point for a process of distress and rumination, which can potentially lead to growth (Calhoun et al., 2010; Tedeschi & Calhoun, 2004). Our results underscore the importance of supporting siblings’ self-efficacy as a resource for restoring their assumptive world-views in the aftermath of a traumatic loss. A stable sense of self-efficacy may facilitate adaptive oscillation between loss- and restoration-oriented activities (Stroebe & Schut, 2001), and constructive thought processes (Gillies & Neimeyer, 2006; Neimeyer, 2019), thereby stimulating cognitive building, which are perceived as important elements that can lead to PTG (Tedeschi & Calhoun, 2004).

A strong belief in one’s own self-efficacy may also explain the siblings’ reported growth in personal strength, which was among the two highest-scoring PTG domains in this study. The finding mirrors the results from bereaved parents in the same project (Titlestad et al., 2022), but contrasts with Johnsen and Afgun’s (2021) study of siblings and friends after the Utøya mass attack, where growth was most evident in relational domains and new possibilities. Other studies from the same project as the present study, suggest that siblings often keep emotions at a distance in interactions with family and friends and primarily rely on themselves to cope with the loss (Dyregrov et al., 2025; Meen et al., 2025). Dyregrov et al. (2025) discuss how this “strong sibling” position can be a result of long-lasting negative family dynamic such as harmful upbringing conditions or conflicts related to the deceased prior to death. These mechanisms may help explain why growth in personal strength appears more pronounced among siblings bereaved by a drug-related death compared to those bereaved by other traumatic deaths, such as terror, which typically does not disrupt family life before the loss.

### 4.3. Strengths and Limitations

This study employed a mixed-methods design, which is essential for enhancing validity when investigating complex phenomena (Stroebe et al., 2003). Recruitment for the END project aimed to capture demographic and socioeconomic diversity, resulting in a sibling sample that was relatively heterogeneous but drawn from a generally homogeneous population of bereaved adults. However, certain characteristics may limit generalizability: the sample was overrepresented by women, participants from southern Norway, and individuals with higher education and above-average household income. Consequently, findings on positive growth may primarily reflect the experiences of a more resourceful subgroup of bereaved siblings (mainly sisters).

To maximize variation, no specific time criterion was applied at death, resulting in a wide range from three months to 30 years. This broad timeframe introduces vulnerability to recall bias, as participants’ ability to accurately remember and report past experiences may diminish over time. Another limitation concerns causality: the design allows the documentation of associations between variables and PTG but cannot establish cause-and-effect relationships. Given the difference in sample size and the exploratory nature of the qualitative data, integrated interpretations should be viewed as tentative.

The structure of the interview guide may also have influenced responses. Reflection on growth was placed at the end, which could have prompted more positive narratives through sequential effects. Conversely, the emotional demands of bereavement interviews may have led to participant fatigue, contributing to the scarcity of growth-related narratives. Additionally, asking whether the death added something new or fostered growth may have been challenging for some participants. None of the authors of this article conducted the interviews, limiting opportunities for targeted follow-up questions.

Consistent with Blix et al. (2015), we excluded the spiritual dimension of the PTGI-SF because religiosity was considered less relevant in the Norwegian context than in the American sample for which the scale was developed (Cann et al., 2010). PTG was therefore assessed using eight items rather than ten. This decision restricts comparability with studies using the full instrument; however, to enhance transparency, we report subscale and item-level results, which may be useful for readers.

## 5. Conclusions

This study provides preliminary insights into posttraumatic growth among siblings bereaved by a drug-related death. The integration of quantitative and qualitative findings shows that growth is possible even in the context of stigmatized loss, but its expression is complex. Survey results indicate that appreciation of life and personal strengths are the most prominent domains of growth, while regression analysis highlights self-efficacy as the strongest predictor when controlling for time since death. Social support did not contribute uniquely to the model. Interview data add depth by illustrating how growth is lived through strengthened family bonds and increased compassion toward individuals in vulnerable situations. These relational changes were less visible in standardized measures, suggesting that narrative accounts capture dimensions of growth that quantitative tools may overlook.

Taken together, the findings suggest that growth in this context is shaped by internal coping resources and relational adaptation rather than external crisis support. They point to the potential value of interventions that strengthen self-efficacy and foster relational bonds within families while addressing stigma that limits access to wider social support. However, the small and relatively homogeneous sample means that these results should be interpreted with caution. Future research should replicate these findings in more diverse samples and use longitudinal designs to explore how internal and relational resources interact over time to sustain growth.

## Figures and Tables

**Table 1:** Survey (*n* = 78) and interview-sample characteristics (*n* = 10) with mean (M), standard deviations (SD), range, and *n* (%).

Variables	Surveys-Sample				Interview-Sample			
	M	SD	Range	*n* (%)	M	SD	Range	*n* (%)
Female sex (*n* = 78/10)				69 (88.5)				7 (70)
Perceived closeness to the deceased (*n* = 78/10)	4.45	0.88	1–4		4.3	0.95	2–5	
Time since death (in months) (*n* = 78/10)	111.59	92.69	3–360		114.10	70.36	19–216	
Age of the bereaved at time of survey (*n* = 76/10)	38.99	11.71	18–67		42.7	9.65	30–61	
Age of the deceased at time of death (*n* = 78/10)	31.19	7.83	17–53		34	4.99	24–41	
Bereaved’ educational level (*n* = 78/10)								
*College/University*				49 (62.8)				8 (80)
*Senior high school*				22 (28.2)				2 (20)
*Primary school*				6 (7.7)				
*Other*				1 (1.3)				
Employment (*n* = 78/10)								
*Full-time job*				48 (61.5)				8 (80)
*Part-time job*				9 (11.5)				
*On sick leave*				5 (6.4)				
*Retired*				2 (2.6)				
*Studying*				5 (6.4)				1 (10)
*Other*				9 (11.5)				1 (10)
Annual household income (*n* = 77/10)								
*Below 250,000*				5 (6.4)				
*250,000–499,999*				16 (20.5)				3 (30)
*500,000–749,999*				16 (20.5)				3 (30)
*750,000–999,999*				25 (32.1)				3 (30)
*1,000,000–1,250,000*				9 (11.5)				1 (10)
*Above 1,250,000*				6 (7.7)				

**Table 2:** Mean (M), Standard deviation (SD), internal consistency estimates, and intercorrelations for all study variables (*n* = 77).

Variables		1.	2.	3.	4.
1.	Time since death	—			
2.	CSS	0.16	α = 0.758		
3.	GSE-SF	0.02	0.35 **	α = 0.861	
4.	PTGI-SF	0.24 *	0.23 *	0.40 **	α = 0.888
M		112 ^a^	24.1	15.4	29.8
SD		92.69	6.2	2.7	9.7

Note: CSS = The Crisis Support Scale; GSE-SF = The General Self-Efficacy Scale; PTGI-SF = Post-traumatic Growth Inventory, ^a^ = months since death, * *p* < 0.05; ** *p* < 0.01.

**Table 3:** Summary of multiple regression analysis predicting: PTGI-SF (*n* = 73).

			Final Model Estimates	
Variables	β_Step1_	β_Step2_	b	95% CI
Time since death	0.24 *	0.23 *	0.02	[0.002, 0.046]
CSS		0.06	0.10	[−0.251, 0.447]
GSE-SF		0.37 **	1.31	[0.534, 2.094]
R^2^	0.057	0.216		
F(1, 75)	4.713 *			
F(2, 73)		7.390 **		

Note: PTGI-SF = The Post-traumatic Growth Inventory; CSS = The Crisis Support Scale; GSE-SF = The General Self-Efficacy Scale; * *p* < 0.05; ** *p* < 0.01.

**Table 4:** The main theme with additional codes describing positive changes.

Theme	Codes
I. Growth in relation to others	(a) Greater sense of closeness with family members
	(b) Increased compassion towards others in vulnerable life situations

**Table 5:** Joint display integrating survey and interview data.

Quantitative Results (*n* = 78)	Qualitative Theme “Growth in Relation to Others” Elaborates Quantitative Results (*n* = 10), Including Illustrative Quotes	Integrated Interpretation
PTGI-SF total: M = 29.8 (SD = 9.7), α = 0.888, subscales: Relating to others = 7.01; New possibilities = 6.16; Personal Strength = 8.32; Appreciation of Life = 8.33. Regression: GSE-SF β = 0.37 **, Time since death β = 0.23 *	Code: Greater sense of closeness: “After he passed away, there have been some concrete changes, like now we have started hugging each other every time we meet…” (ID 54). “We spend a lot of time together compared to other families” (ID 24).	Subscale scores are consistent with relational growth (Relating to Others), as well as existential and resilience-related aspects (Appreciation of Life, Personal Strength). Regression analyses suggest that self-efficacy and time since death may be associated with these changes, which aligns with qualitative accounts of increased openness and intimacy.
CSS is not significant in the regression, despite its correlation with PTG. PTGI-SF item highlights: Changed priorities (M = 4.38)	Code: Increased compassion toward others: “I have become much more empathetic… A substance abuser has many good qualities…” (ID 31). “He wasn’t just a burden… He had a life; he meant something…” (ID 86)	Qualitative accounts of compassion and humility correspond with relational and existential domains but are not reflected in the CSS measure. This may indicate that longer-term compassion is connected to meaning-making and internal resources, rather than to acute crisis support.

* *p* < 0.05; ** *p* < 0.01.

## Data Availability

Restrictions apply to the datasets because of data sensitivity and ethical approvals.

## Abbreviations

The following abbreviations are used in this manuscript:

PTG	Post traumatic growth
END	Drug Death-Related Bereavement and Recovery-project
PGD	Prolonged Grief Disorder
GSE-SF	The General Self-Efficacy Scale-SF
CSS	The Crisis Support Scale
PTGI-SE	Post-traumatic Growth Inventory
